# Integration of Geriatric Education Within the American Board of Emergency Medicine Model

**DOI:** 10.5811/westjem.60842

**Published:** 2023-12-22

**Authors:** Lauren T. Southerland, Lauren R. Willoughby, Jason Lyou, Rebecca R. Goett, Daniel W. Markwalter, Diane L. Gorgas

**Affiliations:** * The Ohio State University Wexner Medical Center, Department of Emergency Medicine, Columbus, Ohio; † Rutgers New Jersey Medical School, Department of Emergency Medicine, Newark, New Jersey; ‡ University of North Carolina School of Medicine, Department of Emergency Medicine, Chapel Hill, North Carolina; § University of North Carolina School of Medicine, UNC Palliative Care Program, Chapel Hill, North Carolina

## Abstract

**Background:**

Emergency medicine (EM) resident training is guided by the American Board of Emergency Medicine Model of the Clinical Practice of Emergency Medicine (EM Model) and the EM Milestones as developed based on the knowledge, skills, and abilities (KSA) list. These are consensus documents developed by a collaborative working group of seven national EM organizations. External experts in geriatric EM also developed competency recommendations for EM residency education in geriatrics, but these are not being taught in many residency programs. Our objective was to evaluate how the geriatric EM competencies integrate/overlap with the EM Model and KSAs to help residency programs include them in their educational curricula.

**Methods:**

Trained emergency physicians independently mapped the geriatric resident competencies onto the 2019 EM Model items and the 2021 KSAs using Excel spreadsheets. Discrepancies were resolved by an independent reviewer with experience with the EM Model development and resident education, and the final mapping was reviewed by all team members.

**Results:**

The EM Model included 77% (20/26) of the geriatric competencies. The KSAs included most of the geriatric competencies (81%, 21/26). All but one of the geriatric competencies mapped onto either the EM Model or the KSAs. Within the KSAs, most of the geriatric competencies mapped onto necessary level skills (ranked B, C, D, or E) with only five (8%) also mapping onto advanced skills (ranked A).

**Conclusion:**

All but one of the geriatric EM competencies mapped to the current EM Model and KSAs. The geriatric competencies correspond to knowledge at all levels of training within the KSAs, from beginner to expert in EM. Educators in EM can use this mapping to integrate the geriatric competencies within their curriculums.

## INTRODUCTION

Emergency medicine (EM) residents have 3–4 years of training to learn an extensive array of skills. This includes the skills needed to care for older patients, who make up 16–20% of their patients.^1^ ^,^ ^2^ The American Board of Emergency Medicine (ABEM) codifies the skills needed for competency in EM in the Model of the Clinical Practice of Emergency Medicine (EM Model) and the 2021 knowledge, skills, and abilities (KSA).^3^ ^,^ ^4^ The EM Model lists clinical presentations and disease types and the KSAs are a list of skills and abilities integral to EM practice. Many residency programs base their curriculums on these documents. However, it is unclear how best to integrate geriatric teaching within these complex curricula.

In 2010 Hogan et al published eight domains with 26 competencies of geriatric education derived from an expert consensus panel that are considered essential learning during EM residency for the care of older adults in the emergency department (ED).^5^ These competencies are also used for categorizing geriatric continuing education for geriatric ED accreditation and have been pivotal to the development of geriatric EM as a subspecialty.^6^ ^,^ ^7^ Despite this guidance, geriatric concepts are still only minimally integrated into resident education.^8^ Without dedicated training, resident knowledge of geriatric competencies is poor.^9^ ^–^ ^11^ But there is currently no guidance on how to integrate the geriatric competencies within an EM residency curriculum.

Our curriculum is based on the EM Model and KSAs. Our goal was to determine whether the geriatric competencies can be covered by an EM Model-based curriculum.

## METHODS

This project is not human subjects research and did not require institutional board review. The study was a descriptive comparison of the 2019 EM Model and the 2021 KSAs to the 2010 geriatric competencies using a consensus-based process. The KSAs include both a description and a level. They are divided into overarching categories (eg, diagnosis, pharmacotherapy, reassessment) which are then divided into steps.^4^ Each step is given a hierarchy in training (with A the highest and E the lowest). Level A is for advanced knowledge or skills. Level B is the minimal competency level for passing EM residency. Levels C, D, and E are skill steps to reach level B.

In the first phase of consensus mapping, two residents (a second-year EM resident and a fourth-year EM/internal medicine resident) and a geriatric fellowship-trained EM attending independently mapped geriatric competencies using Excel (Microsoft Corporation, Redmond WA). They were instructed to first use the search button to look for exact language and then go item by item through the EM Model and the KSAs to map similar language or concepts. For example, the concept of delirium could be described as altered mental status or encephalopathy. A clear association was defined by the team as 1) a keyword match or 2) consensus that it was likely that an emergency physician lecturing/teaching on the EM Model content item would, in normal teaching practices, teach the geriatric competency. If this was not the case, but the geriatric competency could be incorporated under this topic by someone *intentionally* teaching the competencies, this was listed as a suggested area for incorporation. Reviewers were instructed to be generous with mapping during this first round.

If all three or 2/3 agreed, this was considered initial consensus. Any remaining discrepancies were then independently reviewed by another emergency physician with expertise in resident education (former EM program director and current ABEM executive committee member). The full group met and reviewed the final discrepancies until consensus was reached. The consensus tables were then reviewed independently by two more emergency physicians at external residency programs for content validity. A similar process was used for mapping KSAs. Reviewers were blinded to the KSA level (A-E designation).

## RESULTS

### Incorporation into the 2019 EM Model

The EM Model has 963 items. On the first round, 126 items (13% of content) were identified as potential matches, including all of *17.1 Drug and Chemical Classes*. Round 1 consensus was 96.2% (927 items). Table 1 lists the 20 geriatric competencies (77%) included in the 2019 EM Model. Key word matches included competency #6: “*Demonstrate ability to recognize patterns of (physical/sexual, psychological, neglect/abandonment) that are consistent with elder abuse[,]”* which maps to “*Model Content 14.6.1.3 Patterns of Violence/Abuse/Neglect: Intrapersonal Violence: Elder*.” Others were matched by concept, such as competency #11: “*Assess and correct (if appropriate) causative factors in agitated elders such as untreated pain, hypoxia, hypoglycemia, use of irritating tethers (defined as monitor leads, blood pressure cuff, pulse oximetry, intravenous access, and Foley catheter), environmental factors (light, temperature), and disorientation [,]”* which could be incorporated into teaching on *12.14 Nervous System Disorders: Delirium*.

**Table 1.** The geriatric teaching competencies mapped onto the Emergency Medicine Model of Care.

Geriatric competency	Description	EM model item
G1	Generate a differential diagnosis recognizing that signs and symptoms such as pain and fever may be absent or less prominent in elders with acute coronary syndromes, acute abdomens, or infectious processes.	1.1 Abnormal vital signs
		1.2 Pain
G2	Generate an age-specific differential diagnosis for elder patients presenting to the ED with general weakness, dizziness, falls, or altered mental status.	1.3.1 General- altered mental status
		1.3.4 General- ataxia
		1.3.19 General- fatigue/malaise
		1.3.28 General- lightheadedness/dizziness
		1.3.53 General- weakness
		18.3.2 Multisystem trauma- falls
G3	Document consideration of adverse reactions to medications, including drug-drug and drug-disease interactions, as part of the initial differential diagnosis.	1.3.55 General- toxidromes
		17.1 Drug and chemical classes: *entire section*
G4	In patients who have fallen, evaluate for precipitating causes of falls such as medications, alcohol use/abuse, gait or balance instability, medical illness, and/or deterioration of medical conditions.	1.3.4 General- ataxia
		1.3.53 General- weakness
		18.3.2 Multisystem trauma- falls
G5	Assess for gait instability in all ambulatory fallers; if present, ensure appropriate disposition and follow-up including attempt to reach primary care physician.	18.3.2 Multisystem trauma- falls
G6	Demonstrate ability to recognize patterns of trauma (physical/sexual, psychological, neglect/abandonment) that are consistent with elder abuse. Manage the abused patient in accordance with the rules of the state and institution.	14.6.1.3 Patterns of violence/abuse/neglect- elder
G7	Institute appropriate early monitoring and testing with the understanding that elders may present with muted signs and symptoms (eg, absent pain and neurologic changes) and are at risk for occult shock.	1.3.41 General- shock
G8	Assess whether an elder is able to give an accurate history, participate in determining the plan of care, and understand discharge instructions.	12.8.1 Other conditions of the brain- dementia
		14.5.2 Organic psychoses- dementia
		20.4.5.4 Regulatory/legal- consent, capacity and refusal of care
G9	Assess and document current mental status and any change from baseline in every elder, with special attention to determining whether delirium exists or has been superimposed on dementia.	1.3.1 General- altered mental status
		12.8.1 Other conditions of the brain- dementia
		12.14.1 Delirium- excited delirium syndrome
		14.5.2 Organic psychoses- dementia
G10	Emergently evaluate and formulate an age-specific differential diagnosis for elders with new cognitive or behavioral impairment, including self-neglect; initiate a diagnostic workup to determine the etiology; and initiate treatment.	1.3.18 General- failure to thrive
G11	Assess and correct (if appropriate) causative factors in agitated elders such as untreated pain, hypoxia, hypoglycemia, use of irritating tethers (defined as monitor leads, blood pressure cuff, pulse oximetry, intravenous access, and Foley catheter), environmental factors (light, temperature), and disorientation.	12.14.1 Delirium- excited delirium syndrome
G12	Recommend therapy based on the actual benefit to risk ratio, including but not limited to acute myocardial infarction, stroke, and sepsis, so that age alone does not exclude elders from any therapy.	12.11.1.1 Stroke- intracerebral hemorrhagic stroke
		12.11.1.2 Stroke- subarachnoid hemorrhagic stroke
		12.11.2.1 Stroke- embolic ischemic stroke
		12.11.2.2 Stroke- thrombotic ischemic stroke
		20.4.4.1 Health care coordination- advance directives
G14	Prescribe appropriate drugs and dosages considering the current medication, acute and chronic diagnoses, functional status, and knowledge of age-related physiologic changes (renal function, central nervous system sensitivity).	17.1 Drug and chemical classes: *entire section*
G15	Search for interactions and document reasons for use when prescribing drugs that present high risk either alone or in drug-drug or drug-disease interactions (eg, benzodiazepines, digoxin, insulin, NSAIDs, opioids, and warfarin).	17.1 Drug and chemical classes: *entire section*
G16	Explain all newly prescribed drugs to elders and caregivers at discharge, assuring that they understand how and why the drug should be taken, the possible side effects, and how and when the drug should be stopped.	20.1.1.3 Interpersonal skills- patient and family education
G19	With recognition of unique vulnerabilities in elders, assess and document suitability for discharge considering the ED diagnosis, including cognitive function, the ability in ambulatory patients to ambulate safely, availability of appropriate nutrition/social support, and the availability of access to appropriate follow-up therapies.	20.3.2.6 Ethical principles- care of vulnerable populations
		20.4.4.3.1 Healthcare coordination- activities of daily living/functional assessment
G20	Select and document the rationale for the most appropriate available disposition (home, extended care facility, hospital) with the least risk of the many complications commonly occurring in elders during inpatient hospitalizations.	20.4.4.2.3 Healthcare coordination- hospice referral
G21	Rapidly establish and document an elder’s goals of care for those with a serious or life-threatening condition and manage accordingly.	20.4.4.1 Healthcare coordination- advance directives
		20.4.4.2.1 Healthcare coordination- patient identification for palliative care
G22	Assess and provide ED management for pain and key non-pain symptoms based on the patient’s goals of care.	19.3.3 Anesthesia and acute pain management- analgesia
G23	Know how to access hospice care and how to manage elders in hospice care while in the ED.	20.4.4.2.3 Healthcare coordination- hospice referral

*NSAID*, non-steroid anti-inflammatory drug; *ED*, emergency department.

Initial disagreements included whether signs and symptoms were meant to be used to formulate a differential diagnosis for that symptom or to describe management of the symptoms. There was also a question as to whether G11, which discusses “irritating tethers” as a cause of delirium, should be mapped to all procedures such as *19.4.1.4. Nasogastric tube*. The group decided that this would be better encompassed under the EM Model item for delirium. Table 2 lists the six geriatric competencies without a clear fit within the EM Model and suggestions from the team on where to include them.

**Table 2.** Suggestions for teaching the geriatric competencies that do not fit clearly within the Emergency Medicine Model.

Geriatric competency	Description	Suggestions for teaching geriatric competencies without a clear association with EM Model items
G13	Identify and implement measures that protect elders from developing iatrogenic complications common to the ED including invasive bladder catheterization, spinal immobilization, and central line placement.	Could be discussed under *Procedure Domain* or *Practice-based Learning and Improvement: Patient safety and Medical errors*
G17	Document history obtained from skilled nursing or extended care facilities of the acute events necessitating ED transfer including goals of visit, medical history, medications, allergies, cognitive and functional status, advance care plan, and responsible PCP.	No transitions of care, nursing facility, or disposition areas. Could be taught under *Interpersonal and Communication Skills: Intra-departmental relations, teamwork, and collaboration skills*.
G18	Provide skilled nursing or extended care facilities and/or PCP with ED visit summary and plan of care, including follow-up when appropriate.	No transitions of care, nursing facility, or disposition areas. Could be taught under *Interpersonal and Communication Skills: Intra-departmental relations, teamwork, and collaboration skills*.
G24	Assess and document the presence of comorbid conditions (eg, pressure ulcers, cognitive status, falls in the past year, ability to walk and transfer, renal function, and social support) and include them in your medical decision-making and plan of care.	While individual elements listed are in the model (eg, ulcerative lesions: decubitus), the concept of comorbidity in older adults is distinct from disease-oriented items.
G25	Develop plans of care that anticipate and monitor for predictable complications in the patient’s condition (eg, gastrointestinal bleed causing ischemia).	Could be discussed under *Practice-based Learning and Improvement: Patient safety and Medical Errors*.
G26	Communicate with patients with hearing/sight impairment	Could be discussed under *Interpersonal and Communication Skills: Cultural Competency.*

*ED*, emergency department; *PCP*, primary care physician.

### Incorporation into the 2021 Knowledge, Skills, and Abilities

The initial independent mapping resulted in consensus on 84% of the items (179/214). Of the geriatric competencies, 216 (81%) mapped onto KSAs (Table 3). The most common categories were Communication & Interpersonal Skills (CS0), Pharmacotherapy (PT0), and Transitions of Care (TC0). Of the five competencies that did not map directly onto the KSAs, all had mapping items in the EM Model except one. The one competency that did not map directly to any EM Model or KSA was Effects of Comorbid Conditions (G24): “*Assess and document the presence of comorbid conditions (eg, pressure ulcers, cognitive status, falls in the past year, ability to walk and transfer, renal function, and social support) and include them in your medical decision-making and plan of care*.” Incorporating the potential consequences of comorbid conditions is included in KSA PR2: “*Perform the indicated procedure on an uncooperative patient, patient at the extremes of age (pediatric, geriatric), multiple co‐morbidities, poorly defined anatomy, hemodynamically unstable, high risk for pain or procedural complications, sedation required, or emergent indication to perform procedure, and recognize the outcome and/or complications resulting from the procedure*” (KSA Level B). While the geriatrics competency addresses medical decision-making and the KSA address difficult procedures, there is some overlap in the training required.

Of the 63 matches within the KSA, five (8%) mapped onto advanced level A skills (*DX7, Identify obscure, occult, or rare patient conditions*; and *TI6, Develop protocols to avoid potential complications of interventions*). About half (31, 49%) mapped onto required competency skills (Level B), and the remaining 27 (43%) were developing skills (Level C, D or E, 27, 43%) (Table 3).

**Table 3.** The geriatric competencies were mapped onto the 2021 ABEM knowledge, skills, and abilities list.

Geriatric competency	Description	KSA code	Description	Level
G1	Generate a differential diagnosis recognizing that signs and symptoms such as pain and fever may be absent or less prominent in elders with acute coronary syndromes, acute abdomens, or infectious processes.	DX1	Synthesize chief complaint, history, physical examination, and available medical information to develop a differential diagnosis	C
		DX7	Identify obscure, occult, or rare patient conditions	A
		DX8	Construct a list of potential diagnoses based on the chief complaint	D
G2	Generate an age-specific differential diagnosis for elder patients presenting to the ED with general weakness, dizziness, falls, or altered mental status.	DX1	Synthesize chief complaint, history, physical examination, and available medical information to develop a differential diagnosis	C
		DX7	Identify obscure, occult, or rare patient conditions	A
		DX8	Construct a list of potential diagnoses based on the chief complaint	D
G3	Document consideration of adverse reactions to medications, including drug-drug and drug-disease interactions, as part of the initial differential diagnosis.	DX1	Synthesize chief complaint, history, physical examination, and available medical information to develop a differential diagnosis	C
		PT5	Recognize, monitor, and treat adverse effects of pharmacotherapy	B
G6	Demonstrate ability to recognize patterns of trauma (physical/sexual, psychological, neglect/abandonment) that are consistent with elder abuse. Manage the abused patient in accordance with the rules of the state and institution.	LI8	Adhere to processes and procedures to ensure that appropriate agencies are notified in situations that could pose a threat to individual or public health (eg, violence and communicable disease) in accordance with local legal standards	B
		LI10	Adhere to legal and ethical standards to assess and treat patients presenting to the ED	B
		LI11	Advocate for patients vulnerable to violence or abuse in accordance with legal and ethical standards	B
		LI13	Identify patients vulnerable to abuse or and/or neglect	C
G7	Institute appropriate early monitoring and testing with the understanding that elders may present with muted signs and symptoms (eg, absent pain and neurologic changes) and are at risk for occult shock.	DX7	Identify obscure, occult, or rare patient conditions	A
		DS1	Prioritize essential testing	D
		DS2	Determine necessity and urgency of diagnostic studies	E
G8	Assess whether an elder is able to give an accurate history, participate in determining the plan of care, and understand discharge instructions.	CS5	Communicate information to patients and families using verbal, nonverbal, written, and technological skills, and confirm understanding	B
		CS15	Solicit patient participation in medical decision‐making by discussing, risks, benefits, and alternatives to care provided	C
		HP2	Prioritize essential components of a history and physical examination given limited (eg, altered mental status) or dynamic (eg, acute coronary syndrome) situations	B
		TC13	Ensure patient has resources and tools to comply with discharge plan, which may include modifying the plan or involving additional resources (ie, PCP, social work, financial aid) to optimize compliance	B
		TC17	Explain clearly and ensure patient understanding of diagnosis, discharge instructions, and the importance of follow‐up and compliance with treatments.	B
G9	Assess and document current mental status and any change from baseline in every elder, with special attention to determining whether delirium exists or has been superimposed on dementia.	HP6	Identify relevant historical and physical findings to guide diagnosis and management of a patient’s presenting complaint in the context of their baseline condition	B
G10	Emergently evaluate and formulate an age-specific differential diagnosis for elders with new cognitive or behavioral impairment, including self-neglect; initiate a diagnostic workup to determine the etiology; and initiate treatment.	DX1	Synthesize chief complaint, history, physical examination, and available medical information to develop a differential diagnosis	C
		HP2	Prioritize essential components of a history and physical examination given limited (eg, altered mental status) or dynamic (eg, acute coronary syndrome) situations	B
G12	Recommend therapy based on the actual benefit to risk ratio, including but not limited to acute myocardial infarction, stroke, and sepsis, so that age alone does not exclude elders from any therapy.	CS14	Communicate risks, benefits, and alternatives to diagnostic and therapeutic procedures/interventions to patients and/or appropriate surrogates, and obtain consent when indicated	C
		DS4	Review risks, benefits, contraindications, and alternatives to a diagnostic study or procedure	C
		TI8	Assess indications, risks, benefits, and alternatives for the therapeutic intervention.	B
G13	Identify and implement measures that protect elders from developing iatrogenic complications common to the ED including invasive bladder catheterization, spinal immobilization, and central line placement.	DS4	Review risks, benefits, contraindications, and alternatives to a diagnostic study or procedure	C
		PR2	Perform the indicated procedure on an uncooperative patient, patient at the extremes of age (pediatric, geriatric), multiple comorbidities, poorly defined anatomy, hemodynamically unstable, high risk for pain or procedural complications, sedation required, or emergent indication to perform procedure, and recognize the outcome and/or complications resulting from the procedure	B
		PR7	Recognize the indications, contraindications, alternatives, and potential complications for a procedure	D
		TI8	Assess indications, risks, benefits, and alternatives for the therapeutic intervention.	B
G14	Prescribe appropriate drugs and dosages considering the current medication, acute and chronic diagnoses, functional status, and knowledge of age-related physiologic changes (renal function, central nervous system sensitivity).	PT2	Identify relative and absolute contraindications to specific pharmacotherapy	C
		PT5	Recognize, monitor, and treat adverse effects of pharmacotherapy	B
		PT6	Select and prescribe appropriate pharmaceutical agents based on intended eﬀect and patient allergies	C
		PT9	Select, prescribe, and be aware of adverse effects of appropriate pharmaceutical agents based upon relevant considerations such as intended effect, financial considerations, possible adverse effects, patient preferences, institutional policies, and clinical guidelines.	B
G15	Search for interactions and document reasons for use when prescribing drugs that present high risk either alone or in drug-drug or drug-disease interactions (eg, benzodiazepines, digoxin, insulin, NSAIDs, opioids, and warfarin).	PT2	Identify relative and absolute contraindications to specific pharmacotherapy	C
		PT5	Recognize, monitor, and treat adverse effects of pharmacotherapy	B
		PT9	Select, prescribe, and be aware of adverse effects of appropriate pharmaceutical agents based upon relevant considerations such as intended effect, financial considerations, possible adverse effects, patient preferences, institutional policies, and clinical guidelines.	B
		PT10	Conduct focused medication review and identify agents including nutraceuticals and complementary medicines that may be causing an adverse effect	C
		TI6	Develop protocols to avoid potential complications of interventions	A
		TI8	Assess indications, risks, benefits, and alternatives for the therapeutic intervention.	B
G16	Explain all newly prescribed drugs to elders and caregivers at discharge, assuring that they understand how and why the drug should be taken, the possible side effects, and how and when the drug should be stopped.	CS5	Communicate information to patients and families using verbal, nonverbal, written, and technological skills, and confirm understanding	B
		TC17	Explain clearly and ensure patient understanding of diagnosis, discharge instructions, and the importance of follow‐up and compliance with treatments.	B
G17	Document history obtained from skilled nursing or extended care facilities of the acute events necessitating ED transfer including goals of visit, medical history, medications, allergies, cognitive and functional status, advance care plan, and responsible PCP.	CS6	Elicit information from patients, families, and other healthcare members using verbal, nonverbal, written, and technological skills	D
		CS10	Communicate pertinent information to healthcare colleagues in effective and safe transitions of care	C
G18	Provide skilled nursing or extended care facilities and/or PCP with ED visit summary and plan of care, including follow-up when appropriate.	CS10	Communicate pertinent information to healthcare colleagues in effective and safe transitions of care	C
		TC14	Identify patients who will require transfer to a facility that provides a higher level of care and coordinate this transition of care by ensuring communication with the receiving provider, completion of transfer documentation, education of the patient or surrogate the reasons for transfer, consent for transfer, and arrangement of appropriate transportation.	B
		TC16	Use appropriate tools for transitions of care, discharge instructions, prescriptions, follow-up instructions, and any pending diagnostic studies to promote effective care and decrease error	B
G19	With recognition of unique vulnerabilities in elders, assess and document suitability for discharge considering the ED diagnosis, including cognitive function, the ability in ambulatory patients to ambulate safely, availability of appropriate nutrition/social support, and the availability of access to appropriate follow-up therapies.	OB9	Reassess, manage, and prognosticate the course of patients in ED observation status to determine appropriate disposition.	B
		TC13	Ensure patient has resources and tools to comply with discharge plan, which may include modifying the plan or involving additional resources (ie, PCP, social work, financial aid) to optimize compliance	B
		TC18	Correctly determine the appropriate disposition	C
G20	Select and document the rationale for the most appropriate available disposition (home, extended care facility, hospital) with the least risk of the many complications commonly occurring in elders during inpatient hospitalizations.	CS10	Communicate pertinent information to healthcare colleagues in effective and safe transitions of care	C
		OB1	Identify patients appropriate for management in ED observation status	C
		OB9	Reassess, manage, and prognosticate the course of patients in ED observation status to determine appropriate disposition.	B
		TC12	Assign admitted patients to an appropriate level of care	B
		TC14	Identify patients who will require transfer to a facility that provides a higher level of care and coordinate this transition of care by ensuring communication with the receiving clinician, completion of transfer documentation, education of the patient or surrogate the reasons for transfer, consent for transfer, and arrangement of appropriate transportation.	B
		TC18	Correctly determine the appropriate disposition	C
G21	Rapidly establish and document an elder’s goals of care for those with a serious or life-threatening condition and manage accordingly.	CS3	Elicit patients’ reasons for seeking healthcare and their expectations from the ED visit	D
G22	Assess and provide ED management for pain and key non-pain symptoms based on the patient’s goals of care.	ES15	Elicit the patient’s goals of care prior to initiating emergency stabilization, including evaluating the validity of advanced directives	B
G25	Develop plans of care that anticipate and monitor for predictable complications in the patient’s condition (eg, gastrointestinal bleed causing ischemia).	DS4	Review risks, benefits, contraindications, and alternatives to a diagnostic study or procedure	C
		TI6	Develop protocols to avoid potential complications of interventions	A
G26	Communicate with patients with hearing/sight impairment	CS5	Communicate information to patients and families using verbal, nonverbal, written, and technological skills, and confirm understanding	B
		CS7	Consider the expectations of those who provide or receive care in the ED and use communication methods that minimize the potential for stress, conflict, and miscommunication	B
		CS18	Demonstrate interpersonal and communication skills including adjustment of interactions to account for factors such as culture, gender, age, language, disability, that result in the effective exchange of information and collaboration with patients, families, and all other stakeholders.	B

*KSA*, knowledge, skills, abilities; *ED*, emergency department; *NSAID*, non-steroidal anti-inflammatory drug; *PCP*, primary care physician.

## DISCUSSION

The geriatric competencies for EM residency training integrate well within the EM Model and KSAs, with only one competency not having a direct match. Demonstrating this overlap between the suggested subspecialty curriculum and the EM model can help EM educators ensure that the geriatric competencies are incorporated into their curricula. This mapping could also guide the development of board exam questions, lectures, or simulation cases.

The EM Model is very brief, which can make directing education difficult. For instance, training on the EM Model item *18.3 Multi-system Trauma: Falls* is expounded upon in geriatric competency #4: “*In patients who have fallen, evaluate for precipitating causes of falls such as medications, alcohol use/abuse, gait or balance instability, medical illness, and/or deterioration of medical conditions*.” Or another example, KSA *DX1 “Synthesize chief complaint, history, physical examination, and available medical information to develop a differential diagnosis*” can include a discussion of geriatric competency #3 “*Document consideration of adverse reactions to medications, including drug-drug and drug-disease interactions, as part of the initial differential diagnosis.”* They both describe the initial generation of a differential diagnosis, but the geriatric competency adds pharmacology interactions and adverse reactions to be considered in the differential.

A second finding of this study was that the geriatric competencies align with elements required for minimal KSA competency. This implies that different aspects of geriatric care can (and we argue, should) be taught throughout a resident’s training. It also suggests that the geriatric competencies were well developed for the residency level of training and should not be considered “too advanced” or “subspecialty training.” While prior research has evaluated separate geriatric-specific curricula,^9^ ^–^ ^11^ our work shows that geriatric competencies can be integrated throughout a curriculum based on the EM Model and KSAs. As of 2021, there were only 25 geriatric fellowship-trained emergency physicians, which is not enough for every residency program.^12^ Programs without faculty who have no interest or training in geriatrics could also use external training resources such as the online learning modules at https://geri-em.com/ and at the Geriatric Emergency Department Collaborative (https://gedcollaborative.com/online-learning/).

## LIMITATIONS

One limitation of this project was the consensus definitions used. We were unable to find any existing methods to help us define curricular overlap. While we were strengthened by having representation from multiple EM residency programs, other education experts may have a different interpretation of the domains and competencies and how they are typically taught. Additionally, the reviewers were not all attendings and not all geriatric-fellowship trained. Despite this, first-round consensus was very high (84-96%), which suggests shared knowledge among the group. The EM residents involved in this project have since started fellowships in medical education and palliative medicine, demonstrating their passion and additional understanding in these areas.

## CONCLUSION

The geriatric competencies are included within the EM Model and knowledge, skills, abilities list. The competencies provide more detail for education or board questions. We identified areas of overlap where these subspecialty competencies can be emphasized in EM residency curriculums.
